# Real-world diagnostic potential of bacterial biomarkers of canine periodontitis

**DOI:** 10.3389/fvets.2024.1377119

**Published:** 2024-07-23

**Authors:** C. V. Wallis, M. Soltero-Rivera, C. Harvey, R. M. Reynolds, L. J. Carvell-Miller, A. Colyer, T. S. McKee, T. Mills, P. J. Bergman, P. Watson, L. J. Holcombe

**Affiliations:** ^1^Waltham Petcare Science Institute, Melton Mowbray, Leicestershire, United Kingdom; ^2^Department of Surgical and Radiological Sciences, University of California, Davis, CA, United States; ^3^Colin Harvey LLC, Cherry Hill, NJ, United States; ^4^VCA Clinical Studies, Los Angeles, CA, United States

**Keywords:** diagnostic, microbiota, dog, periodontal disease, detection

## Abstract

**Introduction:**

The objective of this study was to investigate the diagnostic potential of bacterial biomarkers by comparing the performance of molecular detection assays with clinical assessments of dog’s oral health performed by veterinarians.

**Methods:**

Supragingival and subgingival plaque samples were collected from 127 client-owned dogs, pre-booked for procedures under general anesthesia, visiting veterinary practices in the United States. DNA was extracted and bacterial biomarkers quantified using quantitative polymerase chain reaction. Gingivitis and periodontitis were recorded by a trained clinician using the Weighted Gingivitis Periodontitis Score which involved assessing the buccal surfaces of 18 teeth while under general anesthesia. Intraoral dental radiographs of the left and right mandibular first molar teeth were also obtained. These data were then used to establish the diagnostic performance of the molecular assay to detect periodontitis.

**Results:**

An initial conscious, visual oral examination performed by the veterinarian identified 67.7% of dogs as having periodontitis, but examination under general anesthesia indicated a higher proportion (86.6%). Analysis of supragingival plaque samples collected by veterinarians from conscious and unconscious dogs demonstrated the assay had an accuracy of 77.7 to 80.9%, a sensitivity of 77.6 to 81.0%, and a specificity of 80.0%.

**Discussion:**

Use of this molecular screening tool in conscious dogs has the potential to improve early periodontal disease detection and support veterinary decision making, ultimately improving the oral health of dogs and consequently their quality of life.

## Introduction

Periodontal disease is one of the most common conditions encountered in primary care veterinary practice but is underdiagnosed. Data from veterinary practices in the United Kingdom and United States, largely based on examination of conscious dogs, indicate diagnosis levels of less than 20% (1, 2). However, studies of dogs under general anesthesia or post-mortem indicate much higher prevalence levels of 44 to 100% (3–7). Also, a study of Yorkshire terriers indicated that the disease starts in dogs as young as 37 weeks of age where 98% of dogs had at least one tooth with early periodontitis (<25% attachment loss) (8). This disconnect between prevalence and diagnosis is because there are little or no outward clinical signs of periodontal disease until late in the disease process, and therefore an accurate diagnosis requires the levels of clinical attachment loss and bone loss to be determined under general anesthesia (9, 10).

Periodontal disease is a group of conditions resulting from the complex interplay between the dental plaque microbial biofilm, the host, and environmental factors (11–13). Early studies of these complex microbial biofilm communities in dogs were limited, with the majority utilizing culture techniques or pertaining to bacterial species associated with health or late-stage periodontal disease in humans (14–19). Later cloning and sequencing studies showed that 80% of bacteria in dog’s mouths were novel, not having been previously identified in the human oral cavity (20). Advances in sequencing technologies and bioinformatics has led to the identification of distinct bacterial communities associated with canine periodontal health and disease (21–26). Recently, application of machine learning methods to these types of data have begun to elucidate potential bacterial biomarkers of canine periodontal disease (27).

Molecular assays, such as quantitative polymerase chain reaction (qPCR), designed to target bacterial biomarkers, have the potential to improve disease detection in clinical settings. In dogs, analysis of subgingival plaque samples from animals with healthy gingiva (*n* = 70), gingivitis (*n* = 69) and early periodontitis (<25% attachment loss, *n* = 66) showed moderate to strong associations between high throughput sequencing data and qPCR assays developed to enable quantification of specific single species of plaque bacteria (27). Application of five machine learning approaches to qPCR data from assays developed to two periodontal disease associated taxa, Peptostreptococcaceae sp. COT-019 and Clostridiales sp. COT-028, estimated a sensitivity (proportion of those with the condition that have a positive result) of 60 to 74% and specificity (proportion of those without the condition that have a negative test result) 68 to 80% (27). In another study, analysis of subgingival plaque samples from 176 dogs, using qPCR assays designed to detect bacterial species associated with human periodontitis, both *Prevotella intermedia* and *Treponema denticola* were present in a greater number of samples and at a higher prevalence in the irreversible group (periodontitis) compared to the reversible (healthy gingiva and gingivitis) group (28). These studies support the use of a qPCR-based approach as an accurate, sensitive, and cost-effective method for the detection of microbial biomarkers associated with periodontal disease.

The objective of this study was to investigate the real-world diagnostic potential of supragingival plaque by evaluating the relationship between periodontal disease parameters and qPCR assays, designed to detect oral bacterial biomarkers of disease. Early detection of disease in conscious dogs, combined with strict proactive oral care regimes at home, and veterinary periodontal treatments if required, could delay the progression of periodontal disease. This would reduce the likelihood of local consequences, such as oronasal fistulas, pathological fractures and, tooth loss, along with systemic changes that have been associated with the disease (29–34).

## Materials and methods

### Study cohort

A total of 127 client-owned dogs were recruited onto the study. Dogs were eligible for the study if they were over 6 months of age and presenting at VCA^™^ Animal Hospitals for pre-booked procedures under general anesthesia. Procedures included professional dental cleaning or requiring other treatment for periodontal disease. Dogs presenting for select other elective procedures, i.e., male neuter, dermatological procedures, small (less than 1 cm) benign mass removals in non-tension areas and internal medicine scoping procedures in stable patients, and that were not immunosuppressed or compromised, were also eligible. Dogs were required to have a complete Weighted Gingivitis Periodontitis Score (WGPS) tooth set on at least one side of the mouth (Table 1). Dogs were excluded if they had oral tumors, chronic ulcerative stomatitis, malocclusions, and gingival hyperplasia affecting more than one tooth on one quadrant of the mouth, and dogs undergoing orthodontic of maxillofacial trauma repair with an intraoral appliance. Dogs that would not allow a conscious oral examination or plaque sampling due to their temperament were also excluded.

**Table 1:** Weighted gingivitis periodontitis score tooth subset (35).

	Tooth Name	Tooth number	Aspect
Maxilla	Third incisor	103, 203	Buccal
	Canine	104, 204	Buccal
	Third premolar	107, 207	Mesial buccal & distal buccal
	Fourth premolar	108, 208	Mesial buccal & distal buccal
	First molar	109, 209	Mesial buccal & distal buccal
Mandible	Canine	304, 404	Buccal
	Third premolar	307, 407	Mesial buccal & distal buccal
	Fourth premolar	308, 408	Mesial buccal & distal buccal
	First molar	309, 409	Mesial buccal & distal buccal

This study was approved by the Waltham Animal Welfare and Ethical Review Body (AWERB) on the 2nd of July 2018 and an addendum submitted and supported 26th of June 2019. At the time of this study veterinary hospitals did not have their own ethical review boards and therefore adhered to AWERB requirements and, veterinarians who were conducting routine veterinary dental treatment followed local regulations. No dogs were anesthetized solely for the purposes of the study. Informed written client consent was obtained which included approval for the pet to participate in the study and for use of their dog’s plaque samples for research purposes. The approval also included access to and sharing with third parties on an anonymized basis the owner’s questionnaire responses and the pets’ medical health records.

### Study design

Eligible dogs, based on the inclusion and exclusion criteria detailed above, were enrolled onto the study either during an initial consultation at the pet hospital or via a telephone call. The veterinary staff completed a medical questionnaire, which incorporated questions on breed, reason for visit, medical conditions and medication including date and duration of most recent (within 6 months) oral antibiotic course or non-steroidal anti-inflammatory drug administration.

On the day of the procedure a veterinary professional performed a physical examination and routine blood work (hematology and biochemistry) to ensure suitability of the dog for general anesthesia as per standard veterinary hospital practice. The veterinarian also performed a conscious visual oral examination to provide a preliminary indication of the dog’s oral health and collected a supragingival plaque sample (see Sample Collection section for details).

During the procedure under general anesthesia the veterinarian collected a second supragingival plaque sample and performed a detailed clinical assessment where the levels of gingivitis (inflammation) and periodontitis (clinical attachment loss and bone loss) were determined. When assessing the level of gingivitis, a subgingival plaque sample was also collected (see Sample Collection section for details). Once all measurements and sample collections were completed, a professional dental cleaning was performed together with other oral treatments if required.

**Figure 1:**
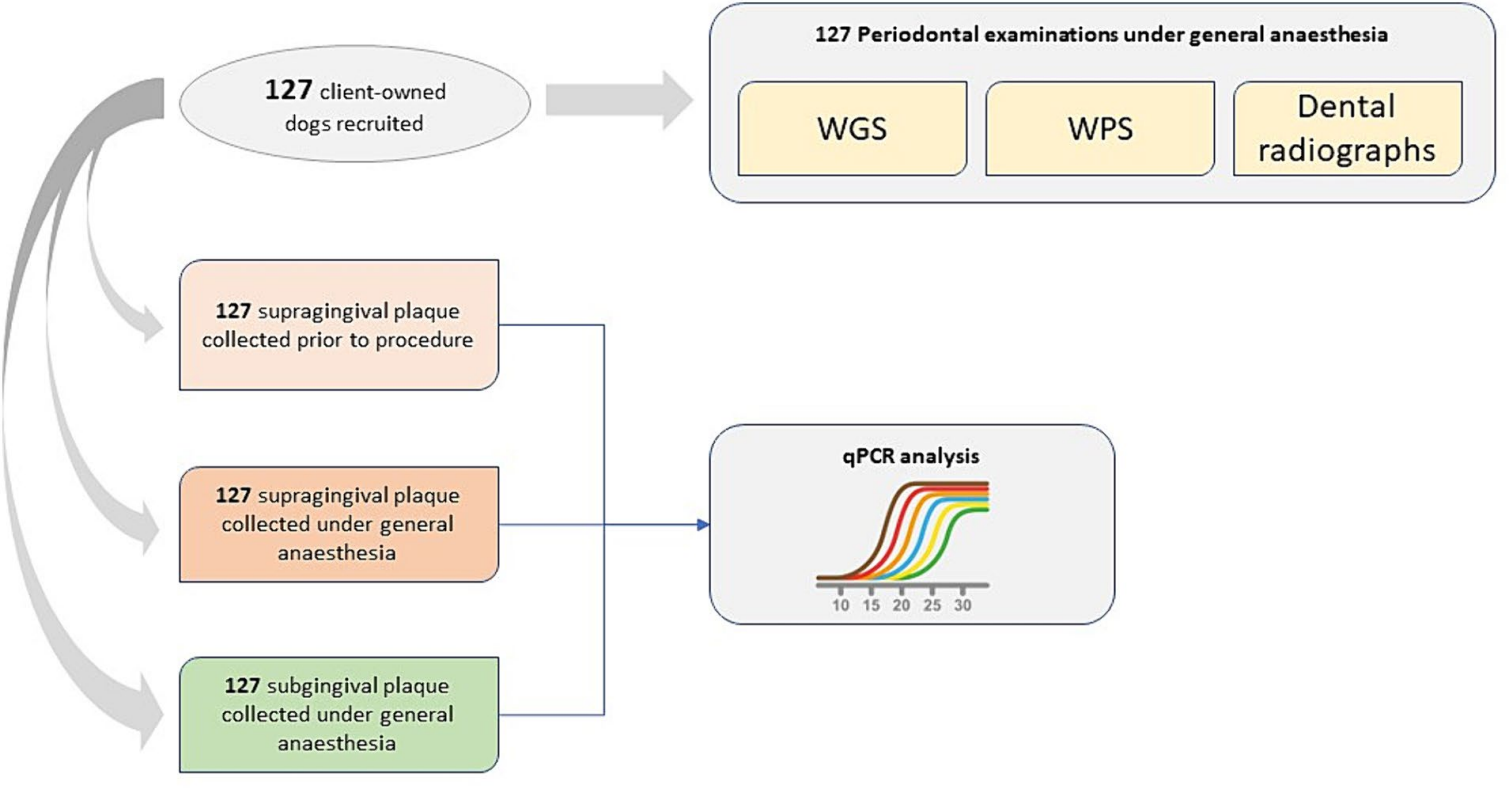
Schematic of clinical data and plaque samples collected by veterinarians across three sampling occasions.

Figure 1 provides a schematic of clinical data and plaque samples collected by veterinarians across the three sampling occasions. The study was managed using an Electronic Data Capture (EDC) system.^1^

### Periodontal examination

Clinical assessments were performed by veterinary staff employed by one of three animal hospitals: VCA Loomis Basin Veterinary Clinic (California), VCA Cordova Dentistry Center (California), VCA Ocean County Animal Hospital (New Jersey). To ensure consistency all received training on dental assessments, scoring and plaque sample collection from one of two Diplomates of the American Veterinary Dental College (AVDC; Maria Soltero-Rivera and Colin Harvey).

Gingivitis and periodontitis were assessed using the WGPS which involved assessing and recording measurements for the buccal surfaces of 18 teeth (30 root sites) for each dog (36) (Table 1). Periodontal examination was undertaken using a blunt-ended periodontal probe (UNC 15 for large breeds and a Michigan O with Williams probe with markings for small to medium sized dogs) and a sharp-tipped dental explorer (#23 shepherd’s hook). All data were captured on an electronic Animal Dental Chart (ADC)^2^ which had been modified to capture WGPS data.

The Weighted Gingivitis Score (WGS) used a gingival bleeding index (Table 2), and the maximum gingivitis score for each root surface was recorded. A weighting factor, circumference of the cemento-enamel junction (CEJ) at each site, was applied to each measurement to account for the size of tooth as described in the original published method (36). The overall WGS score was then calculated by averaging the sum of scores for the left-hand side of the mouth and the sum of scores for the right-hand side of the mouth.

**Table 2:** Gingival bleeding index.

Grade	Description
G0	Healthy, no inflammation visible and no bleeding on probing
G1	Mild gingivitis, inflammation (redness, swelling) visible but no bleeding on probing
G2	Moderate gingivitis, inflamed, delayed bleeding on probing
G3	Severe gingivitis, severe inflammation, prompt profuse bleeding on probing and may be ulceration
G4	Very severe inflammation, blood present prior to probing, severe inflammation and may be ulceration

The Weighted Periodontitis Score (WPS) used the maximum depth in millimeters (mm) from the CEJ to the bottom of the pocket at each root site. The height of the crown (CEJ to top of crown) of one intact maxillary canine tooth was also measured in mm using an endodontic ruler. A weighting factor, root surface area at each site, was applied to each measurement to account for the size of the root as described in the original published method (36). The overall WPS score was then calculated by averaging the sum of scores for the left-hand side of the mouth and the sum of scores for the right-hand side of the mouth. The WPS score was then adjusted for the size of teeth by multiplying by 10 and dividing by the height of the crown of the canine tooth. Missing or mobile teeth, furcation exposure, gingival hyperplasia, tooth resorption and tooth fractures were also recorded if present.

In addition to the measures taken for the study each dog had a complete oral evaluation, whole mouth intraoral dental radiographs and received a professional dental cleaning and other treatment as deemed necessary.

### Dental radiographs

Intraoral dental radiographs of the left and right mandibular first molar teeth (2 radiographs per dog) were obtained and uploaded by VCA^™^ Animal Hospital staff to Antech Imaging Services (AIS^®^).^3^ The radiographs were independently evaluated by two board certified veterinary dentists for severity of alveolar bone loss. If more than one image was available for interpretation the following criteria for selection of the image to be measured were applied: mesial-distal angulation and superimposition of neighboring teeth minimal, distal cusps superimposed, and ventral cortex included. The mandibular first molar teeth were centered in all the images that were measured.

Using the AIS^®^ interface, radiographic measurements were taken of the root length, alveolar bone level and bone loss at the mesial surface of the mesial root and the distal surface of the distal root (2 sites per tooth). Root length was defined as the distance from the CEJ to the apex of the tooth root. More specifically, the root length was measured from the obvious change in angle as seen mesially or distally to represent the CEJ to the mid-point of the apex of that root. If there was a root fracture, with possible overlap of root fragments, and the root fragments were positioned to allow each of the two root fragments to be seen separately, the length of both fragments was measured and then added to represent total root length. Alveolar bone loss was defined as the distance from the CEJ to the alveolar bone crest. In cases of obliquity, foreshortening was confirmed by the appearance of the interproximal bone extending above the CEJ, and in these cases an alveolar bone loss of zero was reported. In cases of increased periodontal ligament (PDL) width, the alveolar bone level was based on the interproximal bone rather than the change in opacity within the PDL space. Percentage bone loss was then calculated as alveolar bone level relative to root length (37). The overall percentage bone loss for each dog was calculated by averaging the four measurements (2 teeth per dog, 2 sites per tooth).

### Overall mouth classification

To determine the reliability of the qPCR assay at detecting periodontal disease an overall mouth classification was assigned to each dog. First, each tooth was assigned as health, gingivitis or periodontitis according to criteria defined by a Diplomate of the European Veterinary Dental College (Table 3) (21). If furcation exposure was recorded, then the tooth was classified as periodontitis irrespective of gingivitis and probing depth scores. An overall mouth classification was then assigned based on the following criteria; health was defined as less than 6 gingivitis and two periodontitis teeth, gingivitis as greater than or equal to 6 gingivitis teeth but less than three periodontitis teeth, and periodontitis greater than or equal to three periodontitis teeth.

**Table 3:** Criteria for assignment of tooth health state according to weight category of the dog.

	Small (<10 kg)	Medium (10–25 kg)	Large (>25 kg)
Health	WGS = 0 and WPS <2 mm (<3 mm on canine)	WGS = 0 and WPS <3 mm	WGS = 0 and WPS <3 mm (<6 mm on canine)
Gingivitis	WGS >0 and WPS <2 mm (<3 mm on canine)	WGS >0 and WPS <3 mm	WGS >0 and WPS <3 mm (<6 mm on canine)
Periodontitis	WGS >0 and WPS ≥2 mm (≥ 3 mm on canine)	WGS >0 and WPS ≥3 mm	WGS >0 and WPS ≥3 mm (≥6 mm on canine)

### Sample collection

Two supragingival plaque samples were collected by a veterinary professional from each dog: one during a conscious oral examination targeting the maxillary canines, premolars and first molar teeth (two swabs; 103–109: Right maxillary third incisor to first molar teeth and 203–209: Left maxillary third incisor to first molar teeth) and one while the dog was under general anesthesia targeting the mandibular canines, premolars and first molar teeth (two swabs; 304–309: Left mandibular canine to first molar teeth and 404–409: Right mandibular canine to first molar teeth). However, the side of the mouth from which each swab was collected was not recorded. The supragingival plaque samples were collected using a Cytosoft^™^ cytology brush (Medical Packaging Corporation, catalog no. CYB-1). This involved gently gliding, while rotating, the brush for 5–10 s across the gingival margin on the buccal surface of the teeth caudal to rostral. Effort was made to avoid the tongue, alveolar mucosa and palatal rugae to reduce contamination of the bristles. The plaque-containing swabs were air dried for approximately 5 min and then placed back into the paper sleeve in which they were supplied. Samples were sent to Antech^®^ Diagnostics at ambient temperature for processing. Processing involved the addition, of 1 mL TE buffer (10 mM Tris-buffer, 1 mM EDTA, pH 8), sufficient to cover the bristles, and then storage at -80°C.

A subgingival plaque sample was also collected by the veterinarian while scoring the level of gingivitis. Subgingival plaque samples were collected from every tooth in the mouth using a periodontal probe. The sterile probe was gently inserted under the gingival margin and swept along the base of the crown. The plaque was removed from the probe by gently agitating in a 0.5 mL Eppendorf containing 300 μL TE buffer. A single whole mouth sample was collected from each dog. Samples were stored at -20°C for up to 20 months prior to transfer to a -80°C storage facility.

### DNA extraction

DNA was extracted from the two supragingival plaque samples (one swab from each collection event representing one quadrant of the mouth) and one subgingival plaque sample. Plaque samples (300 μL aliquot) were mixed by vortexing, Ready-Lyse^™^ Lysozyme solution (1 μL, Epicenter, catalog number R1804M) added to the bacterial suspension, and the sample incubated at 37°C for 18 h. DNA was then extracted from the plaque samples using the Masterpure™ Gram Positive DNA Purification Kit (Epicenter, catalog number MGP04100) according to the manufacturer’s instructions. The quantity of DNA was determined using a Qubit dsDNA High Sensitivity Assay Kit (ThermoFisher Scientific, catalog number Q32851).

### Quantitative polymerase chain reaction

Proprietary qPCR assays were used to amplify a region of the bacterial 16S rRNA gene from the DNA sample. A description of how the assays were designed, tested and selected can be found in earlier publications (27, 38). Each individual 10 μL qPCR assay reaction contained 5 μL TaqMan^™^ Gene Expression Mastermix (Applied Biosystems, United States), 0.5 μL 20x TaqMan^™^ assay (Applied Biosystems, United States), 1 μL DNA and 3.5 μL nuclease-free water. Each final qPCR assay reaction contained 900 nM of each primer and 250 nM of each qPCR probe. Positive controls (16S rRNA clones to uncultured species belonging to the family Peptostreptococcaceae at concentrations of 0.01, 0.0001, 0.000001 ng/μl) and the negative control (TE buffer) were included with each qPCR run. All qPCR assays were performed in triplicate on an ABI^™^ QuantStudio^™^ 7 Flex (Applied Biosystems, United States) machine according to the manufacturer’s instructions. PCR cycle conditions were 50°C for 2 min followed by 95°C for 10 min and then 40 cycles at 95°C for 15 s and 60°C for 1 min. Experimental data were reported as cycle threshold (Ct) values.

The target bacterial species were deemed not detected if all triplicate values were either undetermined or outside the assay limit of quantification (LOQ). If only one or two values were obtained for a triplicate, or the percentage coefficient of variation was >2, the assay was repeated to generate a further three values. If <3 Ct values were obtained the bacterial species was deemed not detected. Providing ≥3 Ct values were obtained the mean Ct value was calculated and then corrected by multiplying by the efficiency value for the assay as determined from a standard curve.^4^ The Peptostreptococcaceae sp. was then normalized to the total amount of bacterial DNA in each sample (Normalized value = Ct Peptostreptococcaceae sp.−Ct total) and then linearized (2^^- normalized value^).

### Statistical methods

Intra class correlation coefficient (ICC) was calculated between the two scorers on the bone loss data, along with the 95% confidence intervals (CI) and *p*-value. A statistically significant agreement between the scorers was concluded when *p*-value ≤0.05, and the following criteria was used for interpretation of the coefficient: < 0.39 low; between 0.40 and 0.59 moderate; >0.60 high. A Bland–Altman graph was plotted to assess agreement between the two scorers. Where the scorers were in significant agreement, their scores were averaged to create one value per dog, and this was used in subsequent analyses.

The diagnostic performance of the assay to detect periodontitis was determined by calculating the number of true positives (TP), true negatives (TN), false positives (FP) and false negatives (FN) by comparing the qPCR result to the overall mouth classification: Health or periodontitis (see methods section “Overall mouth classification”). For the purposes of determining the test performance at detecting periodontitis, results from dogs with gingivitis were excluded. The accuracy (TP + TN/ TP + TN + FP + FN), sensitivity (TP/TP + FN), specificity (TN/TN + FP) and positive predictive values (TP/TP+ FP) and negative predictive values (TN/TN + FN) were estimated with 95% CI (39). The number of dogs required was determined based on a target of 80% (+/−7%) accuracy. This was estimated using the sample size calculation for estimating a binomial proportion (i.e., the accuracy) assuming a normal approximation. This approach calculated the approximate sample size required for the 95% CI of the estimated accuracy (assumed to be 80%) to be within 7%, resulting in 125 animals.

Linear mixed effects models (LMM) were used to compare clinical scores of different regions of the mouth. The absolute difference in WGS or WPS was calculated for pairwise comparisons between quadrants. The absolute differences were the response variable, the pairwise comparison as the fixed effect, and individual animal nested in breed size as the random effect to account for breed size and within animal variability. Age was explored as a random effect, but the model failed to converge due to over complexity. The model residuals were then visually inspected (normal probability plot and plotting residuals against fitted values) to assess whether the model assumptions were upheld. Where the residuals were deemed to be not normally distributed, a log_10_ transformation was applied prior to model fitting. The estimated means, 95% CI and unadjusted *p*-values were obtained, for each pairwise quadrant comparison. One-sided equivalence tests were performed for the mean pairwise comparisons to an upper equivalence limit of 0.5 for WGS and WPS. Comparisons were deemed equivalent when the *p*-value ≤0.05.

Pearson’s correlation coefficient was calculated and tested for significance when exploring relationship between the log_10_ transformed qPCR data (+0.00001 to account for zeros) and clinical data, and between the different quadrants of the mouth for the clinical data. To test the influence of outlying observations, a sensitivity analysis was then performed by removal of samples with WPS >3 (10 samples removed) and the average percentage bone loss being greater than 25 (3 samples removed).

All analyses were performed in R version 4.2.2 (40) using packages ggplot (41), multcomp (42), dplyr (43), ggrepel (44), patchwork (45). All *p*-values were unadjusted due to the nature of the analyses being exploratory.

## Results

### Study cohort

Of the 127 dogs recruited onto the study, the majority were pre-booked for routine professional dental cleaning under general anesthesia (96.1%), and some of which (13 dogs) were also recorded as having halitosis, bleeding or inflamed gums, fractured teeth, infected teeth, or mass/swelling. The remainder (3.9%) were scheduled for a general anesthesia due to fractured or infected teeth or mass/swelling. Most of the dogs (79.5%) did not have any medical conditions, but several were recorded as having disorders such as allergies, endocrine conditions, hepatic and cardiac disease, arthritis, and inflammatory bowel disease. In total, 73.2% of dogs were recorded as receiving medication and the most common types were Apoquel (skin allergies), Bravecto/Simparica (flea and tick), Levothyroxine (hypothyroidism), Trazodone (anxiety), and Heartguard (heartworm).

There were 74 female and 53 male dogs, and their average age was 6.5 years (standard deviation (sd) +/− 3.3; range 1 to 15). The average weight of the dogs was 16.5 kg (+/− 12.6; range 1.9 to 64.9). There were 58 classified as small (< 10 kg), 38 as medium (10–25 kg) and 31 as large (> 25 kg). The average body condition score (BCS) based on the WSAVA 9-point scale [Body-Condition-Score-Dog.pdf (wsava.org)] was 5.3 (+/− 0.7; range 4 to 8). There were 86 dogs deemed to be an ideal bodyweight (BCS score of 5), 10 were considered under conditioned (BCS score of 4) and 31 over conditioned (BCS score of 6 to 8). The majority of dogs (56.7%) were fed solus dry commercial diets, 12.6% received a mixture of dry and wet commercial diets, 6.3% were given a mixture of dry commercial diets & home-prepared cooked food, 7.1% were on prescription diets, 3.1% received a commercial wet diet, and the remaining 3.9% were given other types of food such as raw and freeze-dried diets with or without commercial dry diets. For 10.2% of the dogs the diet was unknown.

### Periodontal health status of dogs

The initial conscious visual oral examination performed by the veterinarian provided a preliminary indication of the dog’s oral health. Of the 127 dogs assessed, 32.3% were classified as gingivitis and 67.7% periodontitis (58 stage 2, 20 stage 3 and 8 stage 4) as defined by the AVDC (AVDC Nomenclature – AVDC.org).

Dental charts capturing WGPS data were obtained for all 127 dogs. Thirty buccal sites were measured per dog which resulted in a total of 2,286 tooth measurements and 3,810 aspect measurements for both WGS and WPS. The average WGS score was 1.5 (sd +/− 0. 7; range 0 to 3.1) and WPS 1.9 mm (+/−1.0; range 0 to 5.8). There were 41 dogs (154 teeth) where a multi-rooted tooth was recorded as having furcation involvement. There were 34 dogs recorded as having mobile teeth (111 teeth). There were also 26 dogs where teeth were recorded as missing (51 teeth).

Although dental radiographs were obtained for 127 dogs (2 teeth per dog), 5.9% could not be located and were therefore not analyzed by the dental experts. There was significant agreement between the dental experts in bone loss measurements with an ICC of 0.9 (95% CI, 0.8, 0.9, *p* < 0.001; Supplementary Figure 1A). This was supported by the Bland–Altman analysis which indicated that 5/102 points (4.9%) fell outside the 95% confidence limits (Supplementary Figure 1B). However, there were 48/120 (40.0%) incidences where one expert classified the bone loss as zero whereas the other recorded a value >0 but less than 14.2%. The average bone loss for the 120 dogs was 5.7% (sd +/−7.5%) and the range was 0 to 38.1%. In total, 103 of the 120 dogs (85.8%) for which radiographs were available were recorded as having bone loss.

### Exploration of potential sampling bias

**Figure 2:**
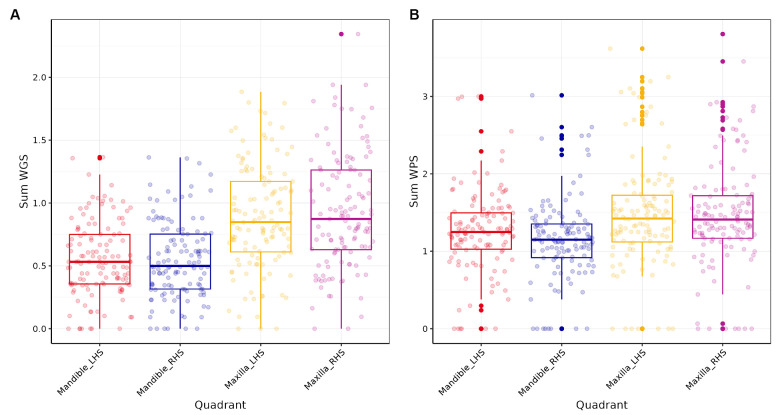
Box plots of the four quadrants of the mouth for **(A)** Weighted Periodontitis Score (WPS) and **(B)** Weighted Gingivitis Score (WGS).

Comparison of the four quadrants of the mouth indicated that WGS and WPS scores were higher for the maxilla than the mandible (Figure 2). The mean WGS for the left-and right-hand side of the maxilla were 0.88 (sd = 0.42) and 0.93 (0.46) and for the left-and right-hand side of the mandible both were 0.55 (0.30). The mean WPS for the left-and right-hand side of the maxilla were 1.47 (0.70) and 1.47 (0.68) and for the left-and right-hand side of the mandible 1.25 (0.56) and 1.15 (0.52). Pearson’s correlation analysis indicated that the WGS and WPS scores were strongly correlated across all quadrants (Table 4). LMM also indicated that WGS and WPS were equivalent across the four quadrants (*p* values <0.001; Supplementary Figure 2). The mean absolute difference in WGS between the left- and right-hand side of the mandible was 0.14 (95% CI: 0.09, 0.19) and the left versus right-hand side of the maxilla was 0.21 (0.16, 0.26). The mean absolute difference in WGS when comparing the maxilla to the mandible ranged from 0.35 to 0.39 depending on the quadrants being compared. The mean absolute difference in WPS between the left- and right-hand side of the mandible was 0.12 (95% CI, 0.02, 0.22) and the left versus right-hand side of the maxilla was 0.14 (0.04, 0.24). The mean difference in WPS when comparing quadrants from the maxilla versus mandible ranged from 0.22 to 0.26. Based on the fact the WGS and WPS values from the four quadrants of the mouth were deemed equivalent, the overall mouth score was used for all subsequent analysis irrespective of which part of the mouth the sample was obtained.

**Table 4:** Pearson’s correlation coefficients for comparisons of the four quadrants of the mouth for weighted gingivitis score (WGS) and weighted periodontitis score (WPS).

Contrast	WGS Pearson’s correlation coefficient (95% CI)	WGS *p* value	WPS Pearson’s correlation coefficient (95% CI)	WPS *p* value
Mandible LHS—Mandible RHS	0.81 (0.74, 0.86)	*p* < 0.001	0.92 (0.89, 0.94)	*p* < 0.001
Maxilla LHS—Maxilla RHS	0.80 (0.72, 0.85)	*p* < 0.001	0.89 (0.85, 0.92)	*p* < 0.001
Maxilla RHS—Mandible RHS	0.79 (0.71, 0.85)	*p* < 0.001	0.84 (0.77, 0.88)	*p* < 0.001
Maxilla LHS—Mandible LHS	0.73 (0.64, 0.80)	*p* < 0.001	0.87 (0.82, 0.91)	*p* < 0.001

### Association between qPCR data and periodontal health status

WGPS data was used to assign an overall mouth classification; 5 (3.9%) dogs were classified as having healthy gingiva, 12 (9.5%) gingivitis, and 110 (86.6%) periodontitis (Supplementary Table 1). Although a complete set of plaque samples were obtained for all 127 dogs, some supragingival samples could not be located following storage, meaning qPCR data was only obtained for 97.2% of supragingival samples (122 from conscious dogs, 124 from unconscious dogs). qPCR data were obtained for all 127 subgingival plaque samples. This resulted in a complete set of three samples for 117 of the 127 dogs (92.1%).

The overall accuracy, sensitivity, specificity and positive/negative predictive values of the qPCR assay were determined by comparing the qPCR results to the overall mouth score (health or periodontitis). The results from the supragingival plaque samples collected from conscious and unconscious dogs indicated overall assay accuracy of 80.9% (CI: 72.3, 87.8%) and 77.7% (68.8, 85.0%) respectively (Table 5). The results from the subgingival plaque samples from unconscious dogs indicated an accuracy of 70.4% (61.2, 78.6%). The sensitivity was highest for the supragingival plaque samples from conscious dogs 81.0% (72.1, 87.8%) and lowest for the subgingival plaque samples from unconscious dogs 70.0% (60.5, 78.4%). The specificity was consistent across all three sampling methods 80.0% (28.4, 99.5%) with the false positive sample always derived from the same dog. Although not included in the determination of the assay performance, the classification of the 12 gingivitis samples was comparable across the supragingival plaque samples collected from conscious and unconscious dogs with the same 7 dogs classified as periodontitis and the same 5 as health based on the qPCR result. The subgingival sample gingivitis results were also in agreement with the supragingival data with the exception of one dog. Of the samples from 104 dogs classified as periodontitis where results were available across all three timepoints (supragingival plaque samples from conscious and unconscious dogs and subgingival plaque samples), 85.6% yielded consistent results. When just comparing the supragingival plaque samples, the agreement was 94.2%.

**Table 5:** Number of true positives (TP), true negatives (TN), false positives (FP) and false negatives (FN) and overall accuracy, sensitivity, specificity, and positive/negative predictive values, with 95% confidence intervals, for three sampling events based on comparison of qPCR result (presence/absence of indicator species) to the overall mouth classification: Health or periodontitis (gingivitis data were excluded).

Sample type	TP	TN	FP	FN	Specificity	Sensitivity	Accuracy	Positive predictive value	Negative predictive value
Supragingival plaque from conscious dog	85	4	1	20	80.0% (28.4, 99.5%)	81.0% (72.1, 88.0%)	80.9% (72.3, 87.8%)	98.8% (93.7, 100.0%)	16.7% (4.7, 37.4%)
Supragingival plaque from unconscious dog	83	4	1	24	80.0% (28.4, 99.5%)	77.6% (68.5, 85.1%)	77.7% (68.8, 85.0%)	98.8% (93.5, 100.0%)	14.3% (4.0, 32.7%)
Subgingival plaque from unconscious dog	77	4	1	33	80.0% (28.4, 99.5%)	70.0% (60.5, 78.4%)	70.4% (61.2, 78.6%)	98.7% (93.1, 100.0%)	10.8% (3.0, 25.4%)

**Figure 3:**
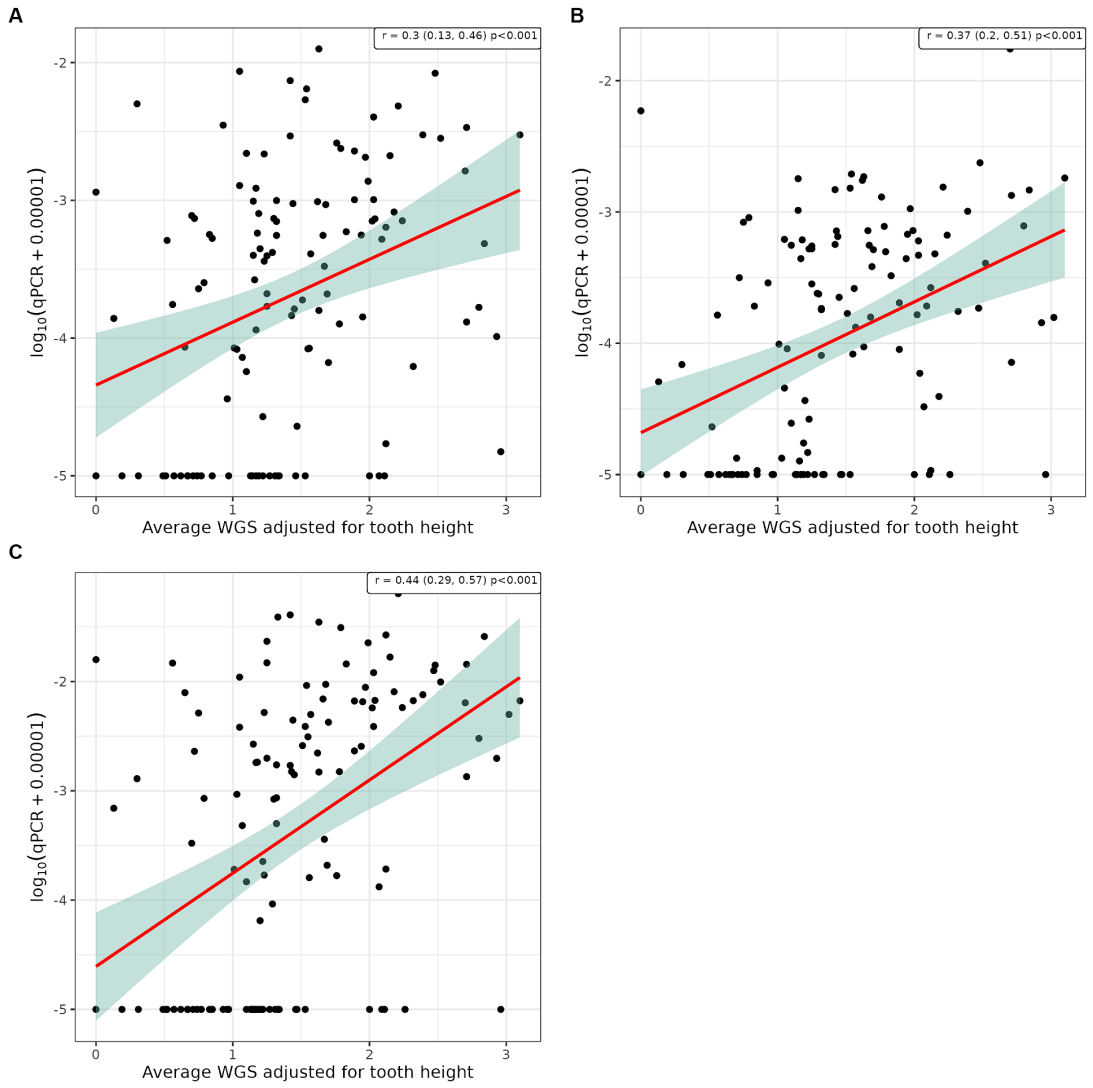
Scatter plot and corresponding regression line (red line) with 95% confidence region (shaded area) of the log_10_ qPCR values versus weighted gingivitis score (WGS) for **(A)** supragingival samples from conscious dogs, **(B)** supragingival samples from unconscious dogs and, **(C)** subgingival samples from unconscious dogs.

**Figure 4:**
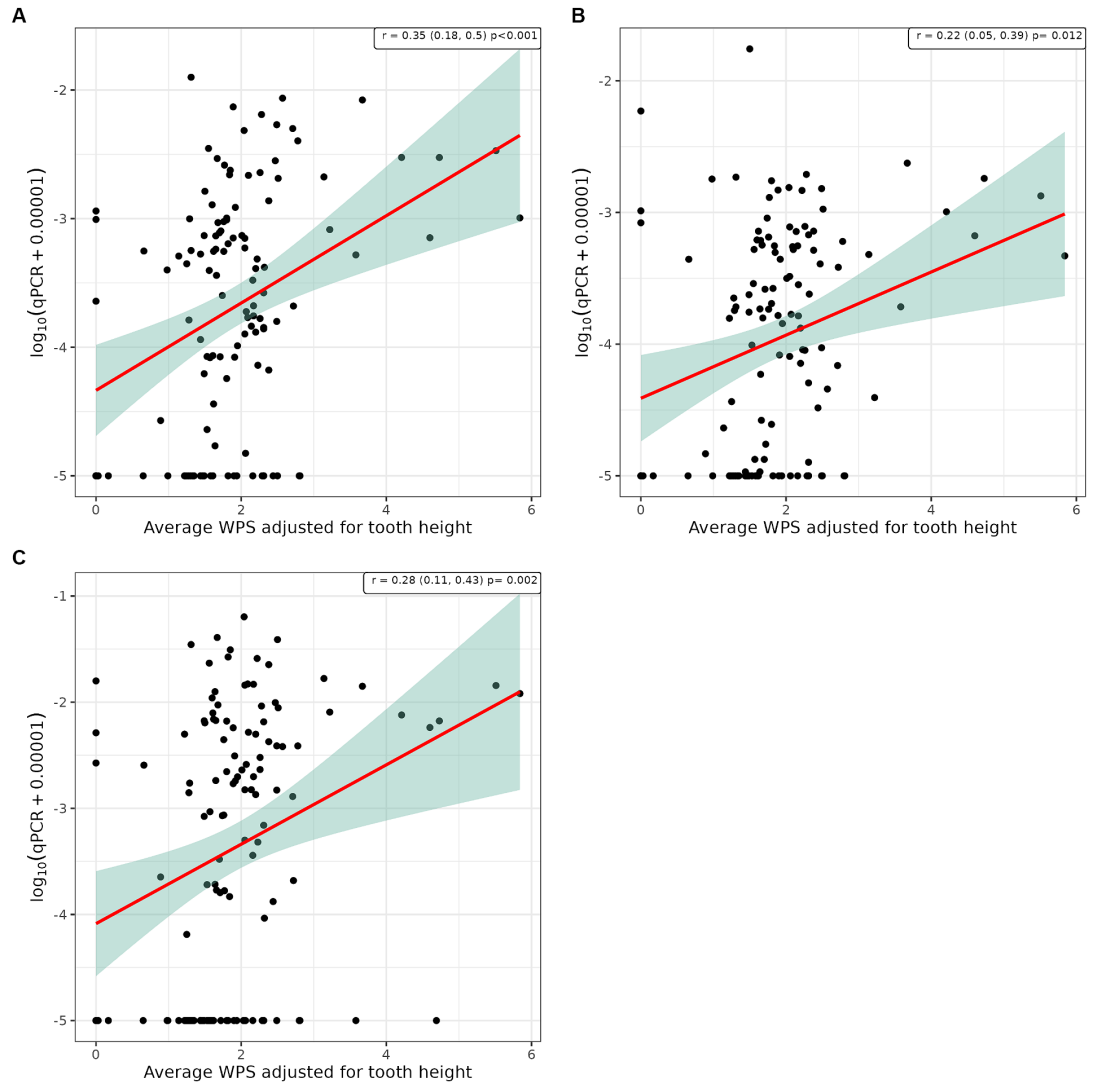
Scatter plot and corresponding regression line (red line) with 95% confidence region (shaded area) of the log_10_ qPCR values versus weighted periodontitis score (WPS) for **(A)** supragingival samples from conscious dogs, **(B)** supragingival samples from unconscious dogs and, **(C)** subgingival samples from unconscious dogs.

**Figure 5:**
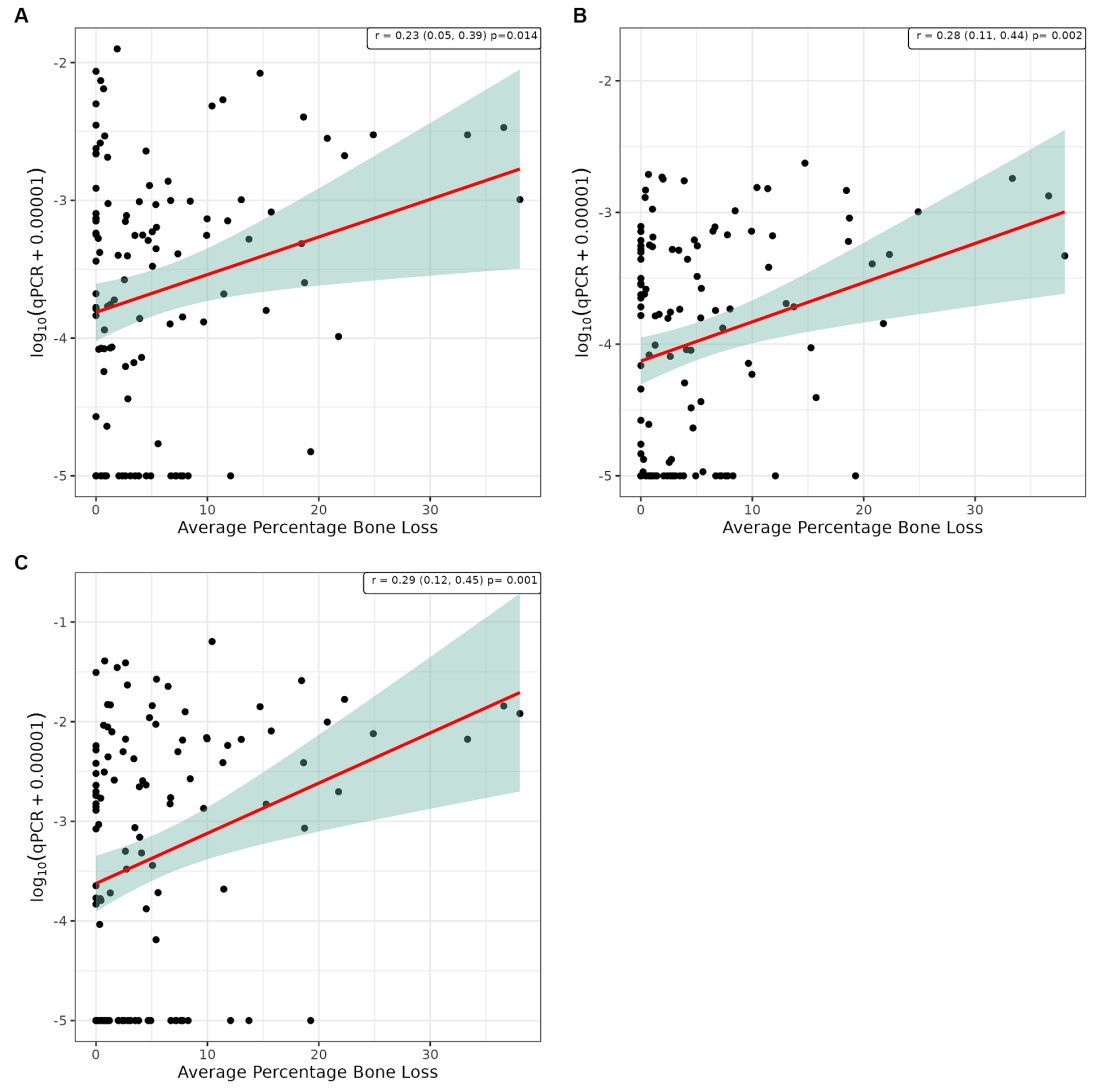
Scatter plot and corresponding regression line (red line) with 95% confidence region (shaded area) for the log10 qPCR values versus bone loss for **(A)** supragingival samples from conscious dogs, **(B)** supragingival samples from unconscious dogs and, **(C)** subgingival samples from unconscious dogs.

Pearson’s correlation analysis was undertaken to explore whether there was a linear relationship between the number of bacterial species, as determined by qPCR, and the severity of periodontal disease. This showed a significant positive correlation between WGS and qPCR values for supragingival plaque samples collected from conscious and unconscious dogs (*r* = 0.30 and 0.37, *p* < 0.001) and subgingival plaque samples (*r* = 0.44, *p* < 0.001; Figure 3). There was also a significant positive correlation between WPS and qPCR values for supragingival plaque samples collected from conscious and unconscious dogs (*r* = 0.35, *p* < 0.001 and *r* = 0.22, *p* = 0.012) and subgingival plaque samples (*r* = 0.28, *p* = 0.002; Figure 4). The sensitivity analysis showed the correlation between WPS and qPCR remained statistically significant for conscious dogs (*r* = 0.21, *p* = 0.024) and for subgingival plaque samples (*r* = 0.21, *p* = 0.021). However, for unconscious dogs the correlation was no longer significant with a coefficient of 0.06 (*p* = 0.551). There was also a significant positive correlation between bone loss and qPCR values for supragingival plaque sample from conscious (*r* = 0.23, *p* = 0.016) and unconscious (*r* = 0.28, *p* = 0.002) dogs and subgingival plaque samples (*r* = 0.29, *p* = 0.001; Figure 5). The sensitivity analysis showed that the qPCR data remained significant with a positive correlation with the bone loss data from the unconscious dogs (*r* = 0.23, *p* = 0.015) and the subgingival plaque samples (*r* = 0.26, *p* = 0.004). However, the samples from the conscious dogs were no longer significant (*r* = 0.18, *p* = 0.061).

## Discussion

The primary objective of this study was to evaluate the potential application of bacterial biomarkers in dental plaque to diagnose periodontal disease in client-owned dogs. The proprietary qPCR assays designed to detect oral bacterial biomarkers of disease (Peptostreptococcaceae sp.) gave an overall accuracy of between 77.7 to 80.9%, depending on whether veterinarians collected samples from conscious or unconscious dogs, and a positive predictive value of 98.8%. The sensitivity was 77.6 to 81.0% and the specificity 80.0%. The sensitivity (70.0%) and accuracy (70.4%) of the test was slightly lower when subgingival plaque samples were utilized, and the specificity was 80.0%. The results from this study are similar to those published based on a retrospective analysis of 70 samples from dogs with healthy gingiva, 69 with gingivitis and 66 with early periodontitis which reported a sensitivity of 74.3% and specificity of 67.5% for qPCR assays targeting Peptostreptococcaceae sp. COT-019 and, 60.0% sensitivity and 80.0% specificity for Clostridiales sp. COT-028 (27).

Although test performance was not reported, another study proposed that *T. denticola* could be used as a biomarker of irreversible canine periodontal disease (28). The study used qPCR assays designed to detect bacterial species associated with human periodontitis to analyze subgingival samples from 176 dogs with reversible (healthy gingiva & gingivitis) and irreversible periodontitis (28). *Prevotella intermedia* and *Treponema denticola* were nearly six times more likely to be detected in the irreversible group compared to the reversible group. In terms of the biggest difference in bacterial prevalence between groups, *T. denticola* was 7.7 times higher in the AVDC-PD3 versus the reversible group and *P. intermedia* 9.3 times higher in the AVDC-PD4 versus the reversible group. Pearson’s correlation analysis indicated that *T. denticola* (*r* = 0.43), *Parvimonas micra* (*r* = 0.43) and *Campylobacter rectus* (*r* = 0.41) were moderately correlated with disease severity in the irreversible group. The Pearson’s correlation analysis was performed using categorical data (i.e., AVDC periodontal disease stage) and are therefore not directly comparable to the current study which was performed using continuous WGPS measures.

The correlation between the qPCR assay results and the WGS, WPS and bone loss measures observed in this study indicates a weak to moderate linear relationship. If the study had been designed and powered to look at correlations between qPCR and the various stages of periodontal disease as defined by the AVDC a higher correlation may have been observed. This lack of strong correlation does not lessen the ability of the test to discriminate between patients that do, or do not, have periodontitis. As such, it suggests a potential application of the test as a screening tool to provide an indication that the condition is present, as opposed to as a strict diagnostic or disease staging tool. Objective screening tools used in conjunction with the findings from a conscious oral visual assessment by a qualified veterinarian can support clinical decision making, including whether a full-mouth examination under general anesthesia, based on periodontal probing and dental radiographs, is required for an accurate diagnosis. Individual risk based on factors such as breed size, breed and age should also be taken into consideration (46). Many owners have reservations about their dog having dental procedures resulting in low rates of compliance with veterinarian recommendations (47). Diagnostic and screening tests provide an objective basis for veterinarians to demonstrate to the pet owner the need for their dog to undergo further investigations and potentially dental treatment and procedures, such as intra-oral dental radiographs and professional dental cleanings under anesthesia. The test also has the potential to promote positive conversations between the veterinarian and owner around effective home-care regimes for proactive management of good oral health.

OraStripdx^™^ is a commercially available test for canine periodontal disease which is described as a simple to use strip that provides a colorimetric signal indicating the levels of thiols in oral fluid. Utilization of thiol-detection tests in routine wellness examinations has been shown to increase the number of dental procedures performed (48). The thiol-detection test was also shown to enhance owner compliance by facilitating discussion with the pet owner about the importance of good oral health and the links with overall wellness of the pet (48). Other studies have also shown that veterinarian recommendations are more likely to be adhered to when they are unambiguous with a clear rationale (49). Several studies have been undertaken to compare OraStripdx^™^ test results to the extent of gingivitis and periodontitis (50). A study of 71 dogs (40 scheduled for dental cleaning or treatment and 31 presumed healthy but not clinically assessed) indicated a strong correlation between the extent of gingival inflammation based on visual oral examinations (*r* = 0.84, *p* < 0.001) and the following measures assessed under general anesthesia; gingival index (*r* = 0.74, *p* < 0.001), periodontal disease stage (*r* = 0.55, *p* < 0.001) and number of periodontal pockets (*r* = 0.44, *p* < 0.001) (50). A follow-up study of 114 dogs performed a visual conscious examination of the oral cavity, followed by the oral-fluid thiol-detection test, prior to a full-mouth detailed examination under general anesthesia (51). The majority of dogs (*n* = 101) had moderate-to-severe active periodontal disease (gingival index ≥2) and 85 had alveolar bone loss. There was a positive association between maximum gingival index and thiol detection test score. However, due to the imbalance in the study population it was not feasible to compare dogs with and without active periodontal disease. Other types of commercial tests, based on profiling the majority of microorganisms within an oral sample using high throughput sequencing, are also available but most utilize knowledge from the human field for interpretation of the results (25, 52).

The thiol-detection test indicated that reliance on visual conscious oral examinations underestimates the number of dogs with active disease (51). Based on the visual assessment, 94 dogs presented with inflammation; however, 113 of the 114 dogs had a positive thiol-detection test result indicating they had active periodontal disease (51). Similarly, in our study 67.7% of dogs were classified as periodontitis based on conscious visual examination by a veterinarian, but 86.6% categorized as periodontitis based on probing the gingiva under general anesthesia, and 85.8% recorded as having bone loss as determined by intra-oral dental radiographs. The relatively small discrepancy in disease classification between conscious and unconscious examinations in the current study is likely due to the fact that the majority of dogs were pre-booked for dental procedures. The tendency to underestimate the presence and severity of periodontal disease and its detrimental effects on the pet’s health has been highlighted previously in a cross-sectional study of 31,484 dogs examined by veterinary practitioners in a chain of private veterinary practices in the United States (53).

Periodontal disease is a site-specific disease in that one or more aspects of the gingiva and teeth may be affected (9). Exploration of potential sampling bias indicated strong correlations between the left- and right-hand side of the mouth and the maxilla and mandible with all four quadrants deemed equivalent. WGPS were generally higher for the maxilla versus the mandible. The maxillary gingival margin has also been shown to display the largest range of thiol concentrations (50). Several studies have reported that periodontal disease in dogs more often affects the maxillary teeth compared to the mandibular teeth (5, 54–57). A number of studies have reported a higher prevalence, and more severe attachment loss, in relation to the maxillary fourth premolar and first molar (4, 6, 56–58). Other studies have also reported the canine teeth as having a high frequency of periodontitis (6, 8, 59). The position of the parotid and zygomatic salivary duct openings has been implicated as a possible reason for the increased levels on the upper 4th premolar (60). For these reasons, and due to the fact that the sensitivity of the test was slightly higher when plaque from the maxillary as opposed to the mandibular teeth of conscious dogs were sampled (81.0% versus 77.6%), the recommendation for optimum test results is to utilize plaque samples from the maxillary teeth from the canine to the first molar.

One of the main limitations of this study, and of the screening tool, was the low level of confidence around the specificity (and consequently the low negative predictive value) due to the small number of healthy dogs recruited. A previous analysis, using the proprietary qPCR assays used in this study, of a more balanced set of banked gingival margin samples with a greater number of healthy dogs (40 healthy dogs and 41 with early periodontitis) indicated a specificity of 65.0% and negative predictive value of 68.4% (21). Data from a greater number of healthy dogs enabled a more accurate determination of the true negative and false positive values, resulting in greater confidence in the specificity and negative predictive value of the assay (Supplementary Table 2). Several studies have shown that a test’s sensitivity and specificity vary depending on disease prevalence (61). Test sensitivity was higher with increased disease severity but with respect to specificity findings have been mixed (61). It is therefore important to ensure that the patient spectrum on which test performance is determined reflects as much as possible the range of animals that a veterinarian will see in practice (62). Although the distribution of oral health states observed in this study of client-owned dogs was likely biased toward dogs with disease, as most were pre-booked for dental procedures, the prevalence of periodontitis (86.6%) was similar to that reported in the literature (i.e., 80% of dogs over the age of three have periodontitis). This is therefore reflective of the level of disease observed in the general dog population (6). Again, due to the small number of dogs without gingivitis it was not possible to investigate the test performance in dogs with active versus inactive periodontitis which may show differing levels of putative periodontopathogens. Periodontal disease is a spectrum ranging from early to severe disease and disease severity has been shown to effect test performance (61, 62). Sensitivity will be lower in animals with early disease, as it is harder to detect subtle changes, and higher at the severe end of the spectrum when the disease is more evident and therefore easier to detect. This phenomenon was supported by the findings from the retrospective analysis of historical subgingival plaque samples from dogs with early periodontitis and late-stage periodontitis where sensitivity was 68.3 and 84.5%, respectively, (Supplementary Table 2). It is therefore important when considering the use of diagnostic tests to account for the disease prevalence and severity for the population in which the test is going to be used.

## Conclusion

Diagnostic tools are routinely used in both human and veterinary settings to provide an indication that a patient is healthy or may have a disease. Results of screening tools, combined with in-depth clinical assessments by appropriately qualified professionals, can support veterinarians in making clinical decisions and providing appropriate care. When determining whether or not to use a diagnostic test it is important to understand the test performance but also take into consideration the benefits and risks. Used appropriately, as a screening test, this molecular tool, based on the detection of bacterial biomarkers of disease in dental plaque, has the potential to improve disease diagnosis rates and support decision making. qPCR is a high-throughput, rapid turnaround method enabling a test result to be obtained within 24–48 h of submission. It also has the potential to promote positive conversations about the importance of effective home-care regimes for proactive management of good oral health. Ultimately this will improve the oral health of dogs leading to an improved quality of life.

## Data availability statement

The original contributions presented in the study are included in the article/Supplementary material, further inquiries can be directed to the corresponding author.

## Ethics statement

The animal studies were approved by Waltham Animal Welfare and Ethical Review Body, Waltham Petcare Science Institute, Melton Mowbray, United Kingdom. The studies were conducted in accordance with the local legislation and institutional requirements. Written informed consent was obtained from the owners for the participation of their animals in this study.

## Author contributions

CW: Conceptualization, Methodology, Project administration, Investigation, Data curation, Formal analysis, Supervision, Validation, Visualization, Writing – original draft, Writing – review & editing. MSR: Conceptualization, Methodology, Investigation, Formal analysis, Writing – review & editing. CH: Conceptualization, Methodology, Investigation, Formal analysis, Writing – review & editing. AC: Conceptualization, Methodology, Formal analysis, Writing – review & editing. LCM: Conceptualization, Formal analysis, Visualization, Writing – review & editing. RR: Project administration, Resources, Data curation, Writing – review & editing. TSM: Conceptualization, Investigation, Methodology, Project administration, Resources, Data curation, Writing – review & editing. TM: Investigation, Resources, Data curation, Writing – review & editing. PB: Conceptualization, Project administration, Funding acquisition, Supervision, Writing – review & editing. LH: Conceptualization, Project administration, Funding acquisition, Supervision, Writing – review & editing. PW: Conceptualization, Funding acquisition, Supervision, Writing – review & editing.

## Footnotes

^1^https://www.castoredc.com

^2^https://animaldentalchart.com

^3^Homepage - Antech Imaging Services

^4^qPCR Efficiency Calculator | Thermo Fisher Scientific - UK
