# Supplementary material for: Real-world diagnostic potential of bacterial biomarkers of canine periodontitis
Source: Front Vet Sci. 2024 Jul 23;11:1377119. doi: 10.3389/fvets.2024.1377119 (PMC11301947; doi:10.3389/fvets.2024.1377119)
Supplement: Supplementary file 1 [file Table_1.DOCX]

**Supplementary Table 1.** Summary of number of teeth classified as healthy, gingivitis, periodontitis and missing, and whether mobile or furcation involvement, and final overall mouth classification for all dogs that had a thorough periodontal examination under general anesthesia.

| Dog ID | Number healthy teeth | Number gingivitis teeth | Number periodontitis teeth | Number missing teeth | Number of teeth with furcation involvement | Number of mobile teeth | Overall mouth classification |
| --- | --- | --- | --- | --- | --- | --- | --- |
| AU1103-001 | 3 | 12 | 2 | 1 |  |  | Gingivitis |
| AU1103-002 |  | 18 |  |  |  | 1 | Gingivitis |
| AU1103-003 | 5 | 13 |  |  |  | 2 | Gingivitis |
| AU1103-004 |  | 9 | 9 |  | 1 | 1 | Periodontitis |
| AU1103-005 | 1 | 15 | 2 |  |  | 2 | Gingivitis |
| AU1103-006 | 1 | 17 |  |  |  |  | Gingivitis |
| AU1103-007 |  | 8 | 10 |  | 5 | 1 | Periodontitis |
| AU1103-008 | 1 | 15 | 2 |  |  |  | Gingivitis |
| AU1103-009 |  | 5 | 11 | 2 | 5 | 3 | Periodontitis |
| AU4013-001 | 18 |  |  |  |  | 4 | Health |
| AU4013-002 | 18 |  |  |  |  |  | Health |
| AU4013-003 | 14 | 4 |  |  |  | 2 | Health |
| AU4013-004 | 6 | 12 |  |  |  |  | Gingivitis |
| AU4013-005 |  | 4 | 14 |  | 12 | 13 | Periodontitis |
| AU4013-006 | 2 | 14 | 2 |  | 2 |  | Gingivitis |
| AU4013-007 | 1 | 17 |  |  |  |  | Gingivitis |
| AU935-001 | 2 | 11 | 5 |  |  |  | Periodontitis |
| AU935-002 | 2 | 4 | 11 | 1 |  |  | Periodontitis |
| AU935-003 |  | 4 | 13 | 1 | 6 | 4 | Periodontitis |
| AU935-004 | 8 | 4 | 6 |  | 2 | 1 | Periodontitis |
| AU935-005 | 4 | 10 | 4 |  |  |  | Periodontitis |
| AU935-006 | 1 | 1 | 16 |  | 2 |  | Periodontitis |
| AU935-007 | 3 | 9 | 6 |  | 2 |  | Periodontitis |
| AU935-008 | 2 | 8 | 8 |  |  |  | Periodontitis |
| AU935-009 |  | 2 | 16 |  | 4 |  | Periodontitis |
| AU935-010 |  | 16 | 2 |  | 1 |  | Gingivitis |
| AU935-011 |  | 9 | 9 |  |  |  | Periodontitis |
| AU935-012 |  | 2 | 16 |  | 3 | 3 | Periodontitis |
| AU935-013 |  | 5 | 13 |  |  |  | Periodontitis |
| AU935-014 | 2 | 15 | 1 |  |  |  | Gingivitis |
| AU935-015 | 4 | 7 | 7 |  |  |  | Periodontitis |
| AU935-016 | 5 |  | 11 | 2 |  |  | Periodontitis |
| AU935-017 | 1 | 3 | 14 |  |  |  | Periodontitis |
| AU935-018 | 2 | 11 | 5 |  |  |  | Periodontitis |
| AU935-019 | 13 | 5 |  |  |  |  | Health |
| AU935-020 | 5 | 3 | 10 |  | 2 |  | Periodontitis |
| AU935-021 |  | 5 | 13 |  | 1 | 1 | Periodontitis |
| AU935-022 |  | 3 | 15 |  | 9 | 2 | Periodontitis |
| AU935-023 |  | 7 | 10 | 1 | 2 |  | Periodontitis |
| AU935-024 |  | 1 | 17 |  |  |  | Periodontitis |
| AU935-025 | 8 | 6 | 4 |  |  |  | Periodontitis |
| AU935-026 |  | 5 | 13 |  | 1 | 4 | Periodontitis |
| AU935-027 | 2 | 3 | 13 |  |  |  | Periodontitis |
| AU935-028 | 12 | 5 | 1 |  |  |  | Health |
| AU935-029 |  | 10 | 7 | 1 |  |  | Periodontitis |
| AU935-030 |  |  | 18 |  |  |  | Periodontitis |
| AU935-031 |  | 2 | 16 |  | 2 | 2 | Periodontitis |
| AU935-032 |  | 8 | 10 |  | 3 | 1 | Periodontitis |
| AU935-033 |  | 3 | 15 |  |  |  | Periodontitis |
| AU935-034 |  | 1 | 17 |  |  |  | Periodontitis |
| AU935-035 |  |  | 14 | 4 | 6 | 10 | Periodontitis |
| AU935-036 |  | 9 | 9 |  |  |  | Periodontitis |
| AU935-037 |  |  | 18 |  |  |  | Periodontitis |
| AU935-038 |  | 1 | 17 |  |  |  | Periodontitis |
| AU935-039 | 4 | 4 | 10 |  | 1 |  | Periodontitis |
| AU935-040 |  |  | 17 | 1 |  |  | Periodontitis |
| AU935-041 | 1 | 8 | 8 | 1 |  |  | Periodontitis |
| AU935-042 | 6 | 8 | 4 |  |  |  | Periodontitis |
| AU935-043 |  | 2 | 16 |  |  |  | Periodontitis |
| AU935-044 |  | 1 | 17 |  |  |  | Periodontitis |
| AU935-045 |  | 1 | 17 |  |  |  | Periodontitis |
| AU935-046 |  | 2 | 14 | 2 | 1 |  | Periodontitis |
| AU935-047 |  | 12 | 6 |  |  | 1 | Periodontitis |
| AU935-048 |  | 12 | 6 |  |  |  | Periodontitis |
| AU935-049 | 4 | 10 | 4 |  |  |  | Periodontitis |
| AU935-050 |  | 9 | 7 | 2 |  |  | Periodontitis |
| AU935-051 |  | 2 | 14 | 2 | 4 | 1 | Periodontitis |
| AU935-052 |  | 13 | 5 |  |  |  | Periodontitis |
| AU935-053 | 1 | 8 | 9 |  |  |  | Periodontitis |
| AU935-054 |  | 6 | 12 |  | 1 |  | Periodontitis |
| AU935-055 |  | 10 | 8 |  |  |  | Periodontitis |
| AU935-056 | 1 | 9 | 8 |  |  |  | Periodontitis |
| AU935-057 |  |  | 16 | 2 | 4 | 6 | Periodontitis |
| AU935-058 |  | 1 | 17 |  |  |  | Periodontitis |
| AU935-059 |  | 2 | 16 |  | 2 |  | Periodontitis |
| AU935-060 |  |  | 18 |  |  |  | Periodontitis |
| AU935-061 |  | 1 | 17 |  | 2 | 2 | Periodontitis |
| AU935-062 |  |  | 18 |  | 12 | 5 | Periodontitis |
| AU935-063 |  |  | 17 | 1 |  |  | Periodontitis |
| AU935-064 | 3 | 7 | 8 |  |  |  | Periodontitis |
| AU935-065 | 5 | 9 | 4 |  |  |  | Periodontitis |
| AU935-066 | 2 | 12 | 4 |  |  |  | Periodontitis |
| AU935-067 |  |  | 18 |  | 7 | 2 | Periodontitis |
| AU935-068 |  |  | 15 | 3 | 4 | 3 | Periodontitis |
| AU935-069 |  | 2 | 16 |  |  |  | Periodontitis |
| AU935-070 |  | 8 | 10 |  |  |  | Periodontitis |
| AU935-071 |  | 14 | 4 |  | 1 |  | Periodontitis |
| AU935-072 |  | 2 | 16 |  |  |  | Periodontitis |
| AU935-073 |  | 11 | 7 |  |  |  | Periodontitis |
| AU935-074 | 1 | 8 | 8 | 1 |  |  | Periodontitis |
| AU935-075 |  |  | 18 |  | 4 | 1 | Periodontitis |
| AU935-076 |  | 12 | 6 |  | 1 |  | Periodontitis |
| AU935-077 |  | 3 | 15 |  | 1 | 2 | Periodontitis |
| AU935-078 |  |  | 18 |  | 1 |  | Periodontitis |
| AU935-079 |  | 17 | 1 |  |  |  | Gingivitis |
| AU935-080 | 1 | 10 | 7 |  |  |  | Periodontitis |
| AU935-081 | 1 |  | 17 |  |  |  | Periodontitis |
| AU935-082 | 2 | 10 | 6 |  |  |  | Periodontitis |
| AU935-083 |  | 1 | 17 |  |  |  | Periodontitis |
| AU935-084 | 1 | 5 | 12 |  | 1 |  | Periodontitis |
| AU935-085 | 2 | 11 | 3 | 2 |  |  | Periodontitis |
| AU935-086 |  | 5 | 13 |  |  |  | Periodontitis |
| AU935-087 | 1 |  | 15 | 2 | 4 | 4 | Periodontitis |
| AU935-088 | 5 | 3 | 10 |  |  |  | Periodontitis |
| AU935-089 | 1 | 7 | 10 |  | 1 |  | Periodontitis |
| AU935-090 | 2 |  | 15 | 1 |  |  | Periodontitis |
| AU935-091 |  |  | 13 | 5 | 3 | 6 | Periodontitis |
| AU935-092 |  | 10 | 8 |  |  |  | Periodontitis |
| AU935-093 |  |  | 18 |  |  | 3 | Periodontitis |
| AU935-094 |  | 12 | 5 | 1 | 1 |  | Periodontitis |
| AU935-095 |  | 1 | 13 | 4 |  |  | Periodontitis |
| AU935-096 | 3 | 6 | 9 |  |  |  | Periodontitis |
| AU935-097 |  |  | 18 |  |  |  | Periodontitis |
| AU935-098 |  |  | 18 |  |  |  | Periodontitis |
| AU935-099 |  |  | 18 |  | 1 | 3 | Periodontitis |
| AU935-101 | 3 | 10 | 5 |  |  |  | Periodontitis |
| AU935-102 | 4 | 8 | 5 | 1 |  |  | Periodontitis |
| AU935-103 | 1 | 13 | 4 |  |  |  | Periodontitis |
| AU935-104 | 2 | 3 | 8 | 5 | 2 | 2 | Periodontitis |
| AU935-105 | 1 | 5 | 12 |  |  |  | Periodontitis |
| AU935-106 |  | 3 | 15 |  | 7 | 1 | Periodontitis |
| AU935-107 |  | 1 | 15 | 2 |  |  | Periodontitis |
| AU935-108 |  |  | 18 |  | 12 | 12 | Periodontitis |
| AU935-109 |  | 7 | 11 |  | 1 |  | Periodontitis |
| AU935-110 |  | 8 | 10 |  |  |  | Periodontitis |
| AU935-111 |  | 4 | 14 |  | 3 |  | Periodontitis |
| AU935-112 | 1 | 5 | 12 |  | 1 |  | Periodontitis |
