# Supplementary material for: Real-world diagnostic potential of bacterial biomarkers of canine periodontitis
Source: Front Vet Sci. 2024 Jul 23;11:1377119. doi: 10.3389/fvets.2024.1377119 (PMC11301947; doi:10.3389/fvets.2024.1377119)
Supplement: Supplementary file 2 [file Table_2.DOCX]

**Supplementary table 2.** A retrospective analysis, using the proprietary qPCR assay, of subgingival and gingival margin plaque samples collected from client-owned dogs visiting veterinary clinics in the USA [21, 22]. Table shows number of true positives (TP), true negatives (TN), false positives (FP) and false negatives (FN) and overall accuracy, sensitivity, specificity, and positive/negative predictive values, with 95% confidence intervals.

| Sample type | TP | TN | FP | FN | Specificity | Sensitivity | Accuracy | Positive predictive value | Negative predictive value |
| --- | --- | --- | --- | --- | --- | --- | --- | --- | --- |
| Supragingival plaque (40 health and 41 early periodontitis, <25% attachment loss) | 28 | 28 | 12 | 13 | 70.0% (53.5%, 83.4%) | 68.3% (51.9%, 81.9%) | 69.1% (57.9%, 78.9%) | 70.0% (53.5%, 83.4%) | 68.3% (51.9%, 81.9%) |
| Subgingival plaque samples (40 health and 58 late-stage disease, >25% attachment loss) | 49 | 28 | 12 | 9 | 70.0% (53.5%, 83.4%) | 84.5% (72.6%, 92,7% | 78.6% (69.1%, 86.2%) | 80.3% (68.2%, 89.4%) | 75.7% (58.8%, 88.2%) |
| Gingival margin plaque samples (40 health and 41 early periodontitis, <25% attachment loss) | 29 | 26 | 14 | 12 | 65.0% (48.3%, 79.4%) | 70.7% (54.5%,83.9%) | 67.9% (56.6%, 77.8%) | 67.4% (.51.5%, .80.9%) | 68.4% (51.3%, 82.5%) |
