# Supplementary figures and images for: Real-world diagnostic potential of bacterial biomarkers of canine periodontitis
Source: Front Vet Sci. 2024 Jul 23;11:1377119. doi: 10.3389/fvets.2024.1377119 (PMC11301947; doi:10.3389/fvets.2024.1377119)

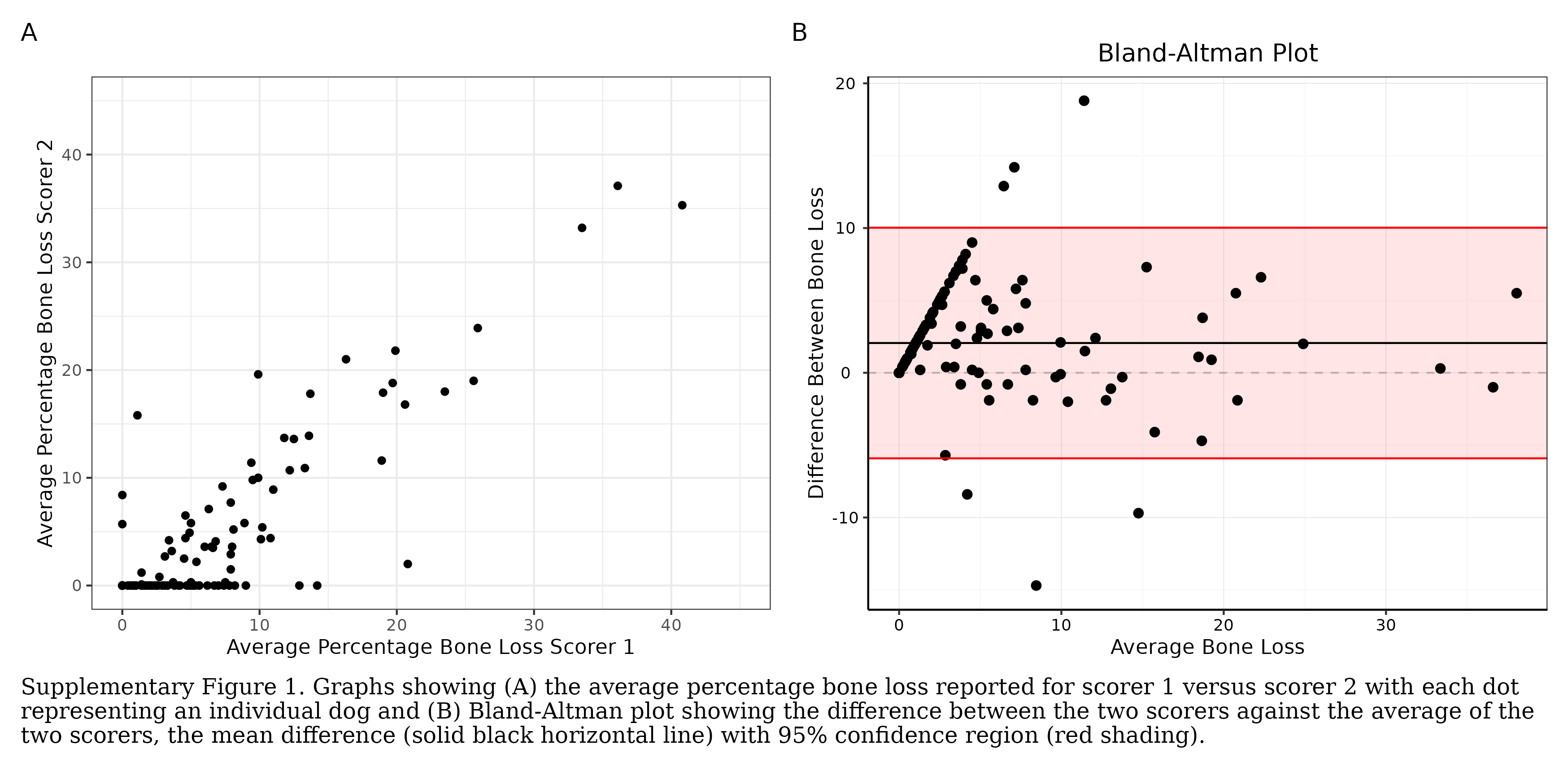

Supplement: Supplementary file 3 [file Image_1.TIFF]

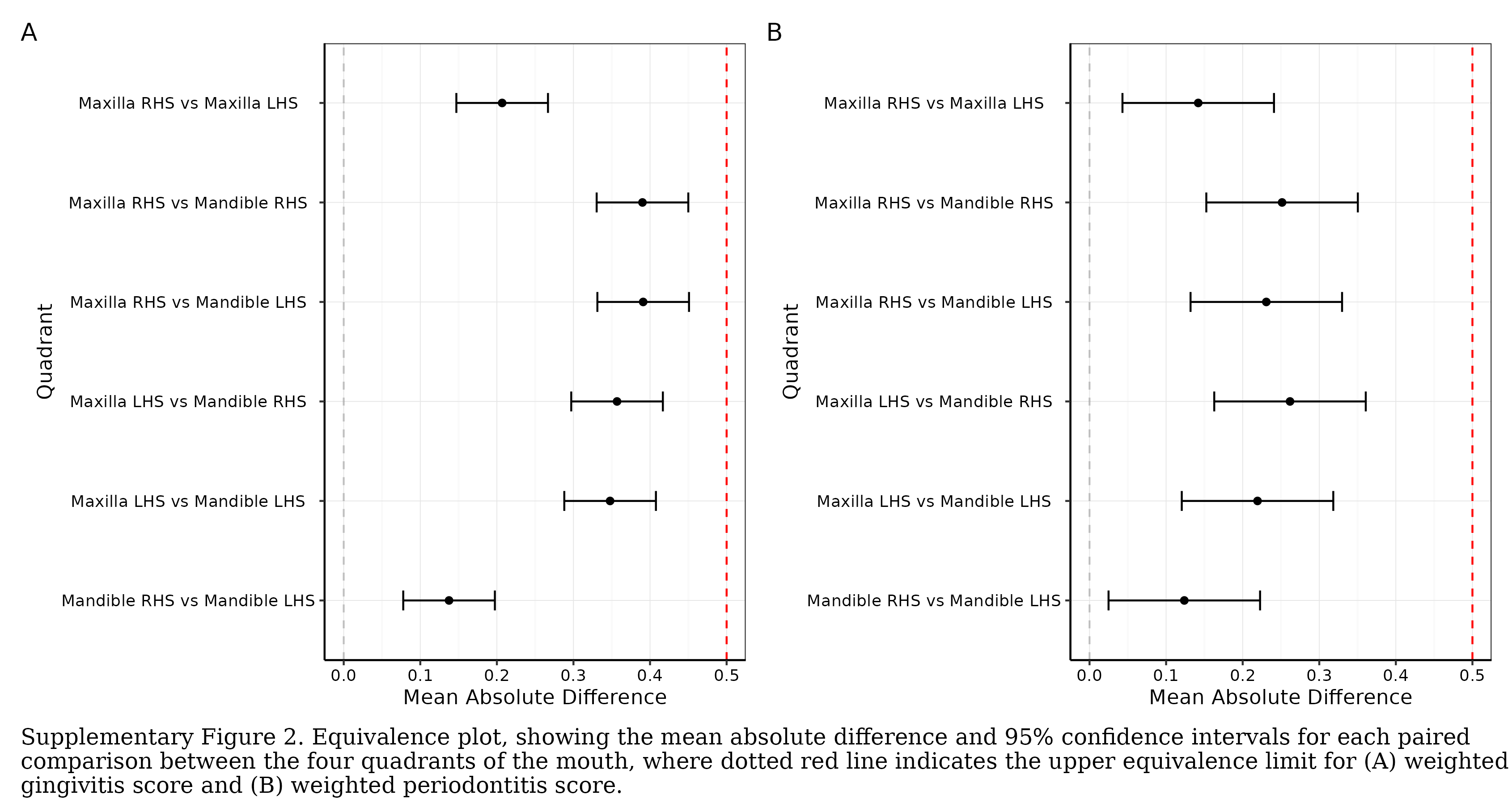

Supplement: Supplementary file 4 [file Image_2.tiff]
